# Increased seroreactivity to proinsulin and homologous mycobacterial peptides in latent autoimmune diabetes in adults

**DOI:** 10.1371/journal.pone.0176584

**Published:** 2017-05-04

**Authors:** Magdalena Niegowska, Alessandro Delitala, Giovanni Mario Pes, Giuseppe Delitala, Leonardo A. Sechi

**Affiliations:** 1 Department of Biomedical Sciences, University of Sassari, Sassari, Italy; 2 Azienda Ospedaliero-Uniersitaria di Sassari, Sassari, Italy; 3 Department of Clinical and Experimental Medicine, University of Sassari, Sassari, Italy; Indian Institute of Technology Delhi, INDIA

## Abstract

Latent Autoimmune Diabetes in Adults (LADA) is a slowly progressing form of immune-mediated diabetes that combines phenotypical features of type 2 diabetes (T2D) with the presence of islet cell antigens detected in type 1 diabetes (T1D). Heterogeneous clinical picture have led to the classification of patients based on the levels of antibodies against glutamic acid decarboxylase 65 (GADA) that correlate with clinical phenotypes closer to T1D or T2D when GADA titers are high or low, respectively. To date, LADA etiology remains elusive despite numerous studies investigating on genetic predisposition and environmental risk factors. To our knowledge, this is the first study aimed at evaluation of a putative role played by *Mycobacterium avium* subsp. *paratuberculosis* (MAP) as an infective agent in LADA pathogenesis. MAP is known to cause chronic enteritis in ruminants and has been associated with autoimmune disorders in humans. We analyzed seroreactivity of 223 Sardinian LADA subjects and 182 healthy volunteers against MAP-derived peptides and their human homologs of proinsulin and zinc transporter 8 protein. A significantly elevated positivity for MAP/proinsulin was detected among patients, with the highest prevalence in the 32-41-year-old T1D-like LADA subgroup, supporting our hypothesis of a possible MAP contribution in the development of autoimmunity.

## Introduction

Latent Autoimmune Diabetes in Adults (LADA) is a slowly developing subtype of immune-mediated diabetes characterized by a combination of clinical features typical to type 2 diabetes (T2D) and reactivity to islet cell antigens commonly detected in type 1 diabetes (T1D). Antibodies (Abs) against glutamic acid decarboxylase 65 (GADA_65_Ab) and protein tyrosine phosphatase IA-2 (IA-2A) are the most conclusive biomarkers [1] that, along with insulin independence period of at least 6 months following disease onset, permit to distinguish LADA from other forms of diabetes. Although general diagnostic criteria has been widely discussed [2, 3], GADA titers remain a benchmark for discrimination between phenotypes recognized among LADA patients presenting clinical characteristics closer to classical T1D or T2D [4].

It is estimated that LADA accounts for almost 10% of newly diagnosed adult-onset diabetes cases [1], therefore its importance in terms of prevalence exceeds that of T1D. Nonetheless, LADA is far less investigated and its etiology remains so far poorly understood. Even though some studies reported increased frequency of predisposing genotypes such as the T2D-associated variant in *TCF7L2* transcription factor [5] or HLA T1D-susceptibility haplotypes [6, 7], major occurrence of less protective genotypes [8] and familial clustering linked to LADA pathogenesis [9], hereditary factors alone cannot explain progression towards β-cell failure in subjects at low genetic risk. Despite its phenotypically heterogeneous nature [10], LADA is a distinct entity frequently associated with other endocrine disorders [11]. The scientific literature describes environmental risk factors related to dietary habits and lifestyle [12, 13, 14, 15, 16], however, in contrast to T1D, scarce information is provided regarding infectious agents putatively contributing to LADA. In this study we focused our attention on a possible role of *Mycobacterium avuim* subsp. *paratuberculosis* (MAP) as an environmental agent at play in LADA pathogenesis. MAP is a globally widespread trigger of a chronic granulomatous enteritis (Johne’s disease) in ruminant animals, able to survive inside host macrophages by overcoming the mechanism of phagocytosis [17] and can be easily transmitted to humans with contaminated water, meat and dairy products [18]. We have previously demonstrated a high prevalence of Abs targeting MAP-derived epitopes and their human homologs of proinsulin (PI) and zinc transporter 8 protein (ZnT8) in T1D at-risk subjects reaching 43% for MAP/PI homologous pair in Sardinian infants [19] and exceeding 62% for MAP/ZnT8 pair in youth from mainland Italy [20]. Interestingly, no significant anti-MAP seroreactivity was associated to T2D [21].

The present study describes humoral responses to the same peptide selection in Sardinian LADA patients with further evaluation based on age-related classification and disease phenotype. We attempted to investigate whether our earlier results may reflect the hypothesis of common factors underlying T2D and LADA etiology [22] and help in identifying the risk for autoimmune comorbidities.

## Methods

### Subjects

Two hundred and twenty three LADA patients (n = 104 men and n = 119 women, mean age 55.22±10.71 years) and 182 age-matched healthy blood donors (HCs; n = 102 males and n = 80 females, mean age 48.13±10.35 years) attending the Transfusion Medicine Department were enrolled in the present study. LADA patients have been referred as a part of a multicentric study, from five Diabetic Units of the island of Sardinia, Italy. The clinical features, autoimmune markers, and progression toward insulin dependence in these patients have been described in detail previously [23]. Any history of autoimmune or inflammatory disorders preceding blood collection from HCs was unknown. Diagnosis of LADA was performed upon analyses for the presence of circulating classical islet autoantibodies (GADA and IA2A), age at onset and period of insulin independence according to the Immunology of Diabetes Society criteria. Venous whole blood was drawn after obtaining written informed consent from all study participants and subjected to plasma separation through sedimentation method for further identification of Abs against MAP peptides and their human homologs deriving from ZnT8 and PI. All patients were free from insulin therapy at the moment of blood collection and none of them developed insulin dependence for at least 6 months following diagnosis. The study protocols were approved by the Bioethical Committee of the University Hospital of Sassari and all methods were performed in accordance with institutional and governmental guidelines and regulations.

### Diabetes-related autoantibodies

Levels of anti-GAD_65_ and anti-IA-2 autoantibodies were determined by radioligand assays employing respective CentAK^®^ anti-GAD65 and CentAK^®^ anti-IA-2 commercial kits (Medipan GMBH, 15827 Dahlewitz, Germany) according to the manufacturer's instruction. GADA titers were used to classify LADA patients in two groups based on a threshold established as previously reported [23], giving 80 subjects with high GADA values assigned to LADA1 subtype and 118 individuals with low GADA levels grouped as LADA2 subtype. Relative data was not available for 25 subjects.

### Peptide antigens

Peptides MAP2404c_70-85_ (RGFVVLPVTRRDVTDV), MAP1,4αgbp_157-173_ (GTVELLGGPLAHPFQPL), MAP3865c_133–141_ (LAANFVVAL) and MAP3865c_125-133_ (MIAVALAGL) along with their respective human-derived homologs PI_46-61_ (RGFFYTPKTRREAEDL), PI_64-80_ (GQVELGGGPGAGSLQPL), ZnT8_186-194_ (VAANIVLTV), and ZnT8_178-186_ (MIIVSSCAV) were synthesized at >85% purity (LifeTein, South Plainfield, NJ 07080, USA) confirmed by HPLC. After reconstitution to 10mM concentration in sterile DMSO, the peptides were stored at -20°C in single-use aliquots for further assays.

### Enzyme-linked immunosorbent assay

Abs specific for MAP1,4αgbp/PI, MAP2404c/PI and MAP3865c/ZnT8 homologous epitope pairs were detected in plasma samples by indirect enzyme-linked immunosorbent assays, as described previously [24]. Each test included a highly responsive serum as a positive reference for data normalization with Abs reactivity set at 1,0 arbitrary units (U/ml) and a negative control to which serum was not added. Optimal positivity thresholds were established at 0.74–0.80 U/ml for MAP/PI and 0.81–0.86 U/ml for MAP/ZnT8 antigen pairs based on the receiver operating characteristic (ROC) curves with specificity set at 90% for all peptides. Mann-Whitney *U* test (95% CI, two-tailed) and Fisher’s exact test were applied to determine statistical significance of the data using Graphpad Prism Version 6.02 (GraphPad Software Inc., La Jolla, CA 92037, USA). Variances of separated groups of LADA1 and LADA2 patients along with HCs were compared employing one-way ANOVA on ranks.

## Results

**Fig 1:**
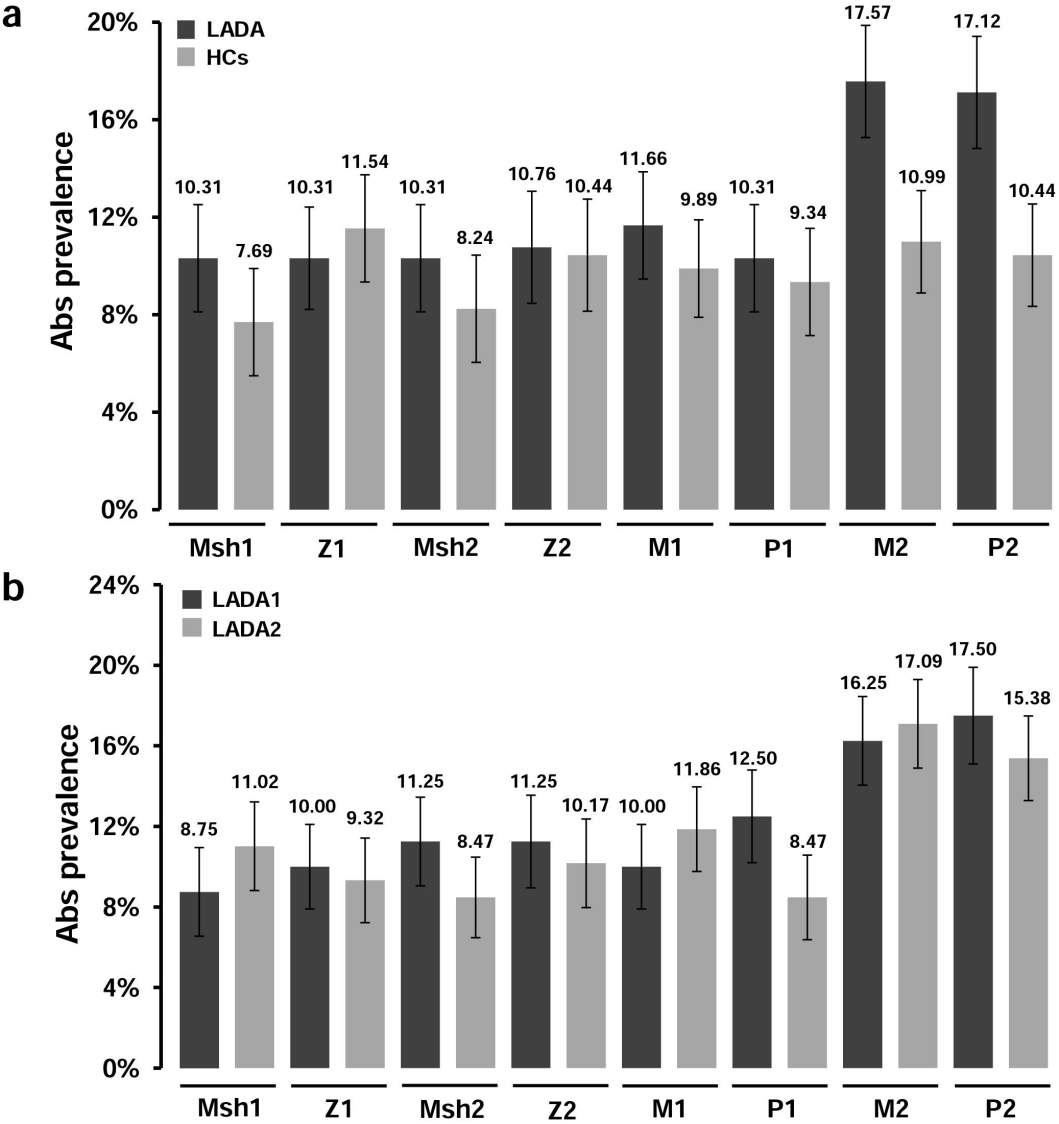
Prevalence of Abs against MAP, proinsulin and ZnT8 homologous epitopes in LADA patients and HCs. The histograms represent percentages of subjects with Abs positivity to selected epitopes upon single-antigen analysis; sera were tested in duplicate for their reactivity against plate-coated peptides: MAP3865c_133–141_ (Msh1); ZnT8_186-194_ (Z1); MAP3865c_125-133_ (Msh2); ZnT8_178-186_ (Z2), MAP1,4αgbp_157-173_ (M1); PI_64-80_ (PI1); MAP2404c_70-85_ (M2); PI_46-61_ (PI2). **(a)** Dark bars represent LADA patients; light grey bars correspond to HCs. **(b)** Abs prevalence among LADA 1 (dark bars) and LADA 2 (light grey bars) patient groups. Relative percentages and standard deviations are reported for each bar.

**Fig 2:**
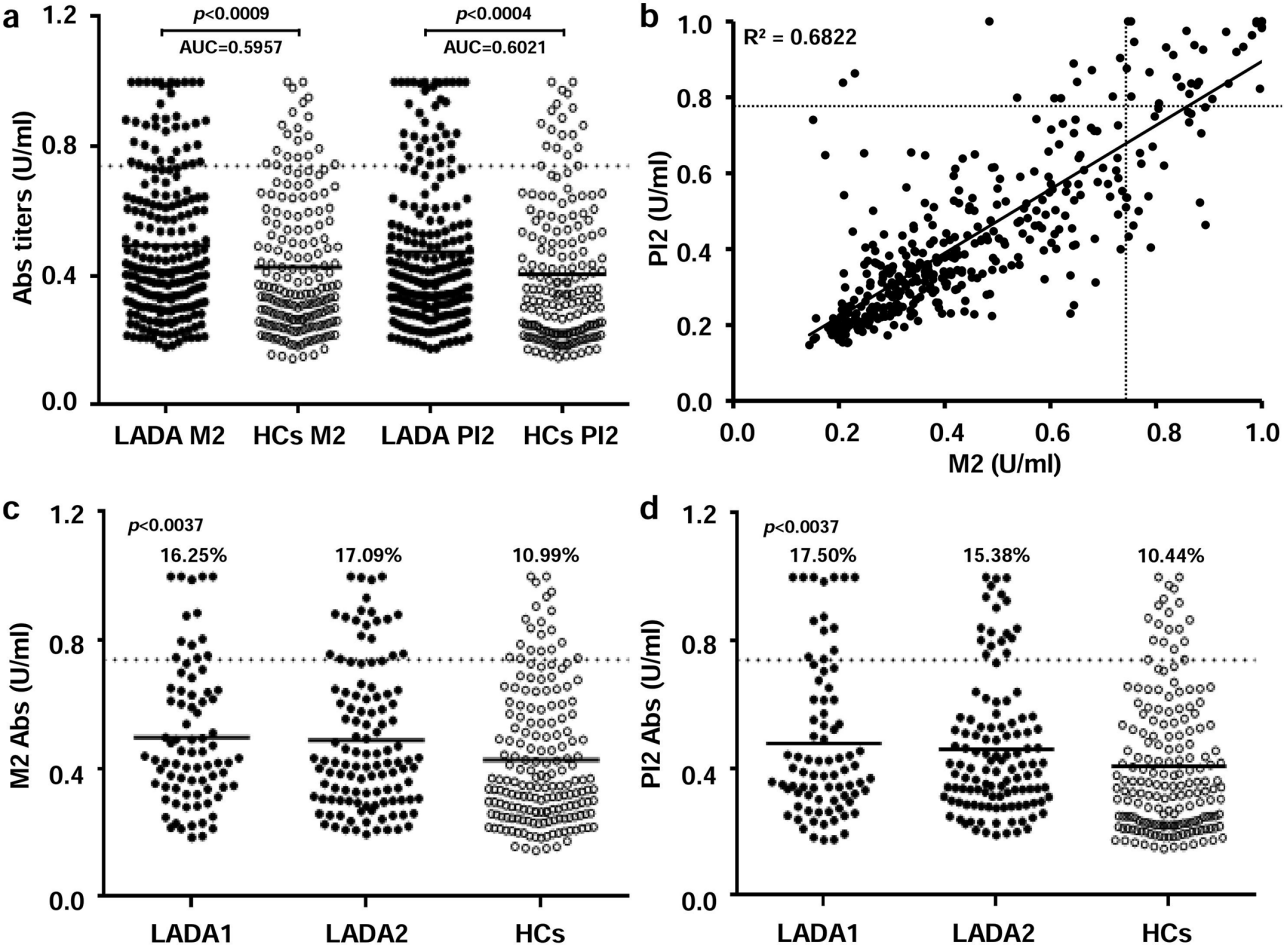
Prevalence and correlation of Abs specific for MAP2404c_70-85_/PI_46-61_ peptide pair in LADA patients and HCs. **(A)** Distribution of Abs values for MAP2404c_70-85_ (M2) and PI_46-61_ (PI2) antigens. Horizontal bars specific for LADA and HCs groups correspond to means. AUC and *p* values (CI 95%) are indicated above the distributions. **(B)** Correlation between Abs recognizing MAP2404c_70-85_ and PI_46-61_ homologous epitopes in LADA subjects and HCs. Each dot represents Abs detected in one sample; in both graphics the dotted lines indicate positivity thresholds established for each assay based on the ROC analysis. **(C)** Distributions relative to Abs titers of M2 and **(D)** PI2 in LADA 1 and LADA 2 patients with HCs as reference; *p* values calculated by Kruskal-Wallis test as well as percentages of Abs prevalence in each group are reported above the distributions. Only statistically significant results are presented.

After a single-peptide analysis, we did not find significant differences in responsiveness to the analyzed antigens between LADA subjects and HCs with the exception of MAP2404c_70-85_/PI_46-61_ pair (Fig 1), for which Abs prevalence characterized by high statistical significance exceeded 17% in LADA patients and 9% among HCs (*p*<0.0009 for MAP2404c and *p*<0.0004 for the homologous PI, Fig 2a). Fisher’s exact test performed based on the numbers of positive and negative subjects further confirmed the significant results when comparing LADA patients and HCs (*p*<0.028 for MAP2404c_70-85_ and *p*<0.039 for PI_46-61_). Moreover, a high degree of correlation (R^2^ = 0.68; Fig 2b) was detected between Abs titers recognizing the two homologs in either patients or HCs suggestive of cross-reactivity.

Responses to the six remaining peptides were distributed evenly among LADA subjects at the level of 10.31–11.66%. HCs displayed somewhat lower Abs positivity towards both MAP-derived peptides homologous to ZnT8 accounting for 7.69%, whereas percentages of anti-ZnT8 Abs were similar to those observed in the patient group. Similarly, prevalence of Abs directed against MAP1,4αgbp_157-173_/PI_64-80_ epitope pair among HCs remained slightly below 10%. Analysis of separated groups defined by high and low GADA titers (LADA1 and LADA2, respectively) confirmed the above mentioned results with respect to HCs, however no differences in mean values or Abs prevalence were found between the two disease phenotypes (Fig 2c and 2d). In fact, MAP2404c_70-85_/PI_46-61_ homologs were recognized by 15.00–16.25% of patients in each LADA group (*p*<0.0037), whereas immune reactivity towards the other selected peptides appeared leveled off at 8.47–12.50% (Fig 1b).

Data relative to other islet autoantibodies were available for 188 patients; among them 43 individuals resulted positive to IA-2A, of which 12 (30.23%, Table 1) had anti-MAP Abs and those directed against MAP2404c_70-85_/PI_46-61_ epitopes were detected in 9 subjects (25.58%). IA-2A-negative patients showed 18.71% of general anti-MAP Abs prevalence that decreased to 16.77% when only the statistically significant pair of homologs was considered. After division of patients in disease subtypes, we obtained 8 IA-2A-positive LADA1 and 35 LADA2 patients among which 3 and 10 subjects, respectively, with anti-MAP/PI Abs. Patients without elevated IA-2A titers had Abs against MAP/PI antigens in 13 cases for LADA1 and 15 for LADA2 (19.70% and 18.99%, respectively).

**Table 1:** Reactivity MAP, ZnT8 and PI antigens in relation to IA-2A titers.

	IA-2A	Patients	Anti-MAP Abs	M2/PI2 Abs*
**LADA**	+	43	12 (30.23%)	9 (25.58%)
	-	145	30 (18.71%)	28 (16.77%)
**LADA1**	+	8	3 (37.50%)	3 (37.50%)
	-	66	13 (19.70%)	12 (18.18%)
**LADA2**	+	35	10 (28.57%)	8 (22.86%)
	-	79	15 (18.99%)	12 (15.19%)

*Antigens for which statistically significant values were obtained.

74 LADA1 and 114 LADA2 subjects with complete clinical data were selected from the study population and further classified in four age groups: 32–41, 42–51, 52–61 and ≥62 years, for which we calculated Abs prevalence either to all peptides together or to the homologous pair MAP2404c_70-85_/PI_46-61_ separately (Table 2). Seroreactivity was evaluated between patients and HCs as well as in distinct LADA phenotypes with regard to age at onset and at the time of blood collection. The youngest and the oldest patients were both diagnosed with LADA1 at 32 and 77 years old, respectively. In all disease groups, the highest reactivity to at least one of the analyzed epitopes was registered in the youngest individuals, even though only slightly lower prevalence was observed after 61 years for LADA2; the weakest responses corresponded to the 42–51 age range except for LADA1 for which the lowest seroreactivity occurred in the oldest subjects. When Abs against only MAP2404c_70-85_/PI_46-61_ homologs was taken into account, we assigned the highest prevalence to the 32–41 group for LADA1 and ≥61 year old patients for LADA2; for both phenotypes quite high positivity with significant values was maintained in the 52–61 group (*p*<0.0001). Independently from the analyzed peptides, HCs presented a progressive loss of Abs with age.

**Table 2:** Seroreactivity to MAP, ZnT8 and PI epitopes in different age groups and during insulin independence period. Percentages of patients positive to the analyzed epitopes are reported along with the relative numbers of seroreactive subjects given in brackets. Mann-Whitney *U* test was employed to calculate statistical significance.

Age group (years)	LADA patients		LADA 1		LADA 2		HCs
	Onset^a^	Sample collection^b^	Onset	Sample collection	Onset	Sample collection	Sample collection
	**Abs prevalence for 8 peptides (%)**						
**32–41**	34.37 (11)	32.26 (10)	40.00(6)	45.45 (5)	29.41 (5)	27.78 (5)	38.46 (20)
**42–51**	8.51 (4)	17,78 (8)	11.11 (2)	20.00 (4)	6.89 (2)	16.00 (4)	18.46 (12)
**52–61**	25.93 (21)	21.43 (12)	25.93(7)	23.53 (4)	25.93 (14)	20.51 (8)	10.64 (5)
**>61**	21.43 (6)	20.69 (12)	7.14 (1)	11.54 (3)	33.33 (5)	27.28 (9)	11.12 (2)
	**Abs prevalence for MAP2404_c70-85_/PI_46-61_ (%)**						
**32–41**	25.00 (8)	22.58 (7)	33.33 (5)	36.36 (4)	17.65 (3)	16.67 (3)	21.15 (11)
**42–51**	8.51 (4)	17,78 (8)	11.11 (2)	20.00 (4)	6.89 (2)	16.00 (4)	10.77 (7)
52–61*	23.46 (19)	17.86 (10)	25.93 (7)	23.53 (4)	22.22 (12)	15.38 (6)	6.38 (3)
**>61**	21.43 (6)	20.69 (12)	7.14 (1)	11.54 (3)	33.33 (5)	27.28 (9)	0.00
**Insulin-free period**^c^	**Abs prevalence for MAP2404_c70-85_/PI_46-61_ (%)**						
**≤12**	17.39 (4)	-	14.28 (1)	-	18.75 (3)	-	-
**13–24**	23.53 (4)	-	0.00	-	33.33 (4)	-	-
**25–36**	18.19 (2)	-	16.67 (1)	-	20.00 (1)	-	-
**37–48**	17.10 (13)	-	18.75 (6)	-	15.90 (7)	-	-
**>48**	14.28 (2)	-	25.00 (1)	-	10.00 (1)	-	-

* Statistically significant.

^a^ Samples collected at the time of LADA diagnosis.

^b^ Samples collected during a control medical visit following LADA onset.

^c^ Expressed in months.

Clinical history regarding insulin-free period after LADA diagnosis was complete for 142 patients. Most of them (n = 127; 63.68% with LADA2 phenotype) required insulin therapy within 4 consecutive years and only 23 subjects undergone insulin treatment within 12 months following LADA onset (69.57% with LADA2). Differences in anti-MAP positivity between periods of insulin independence reached 1% until 48 months when both LADA phenotypes were analyzed together (Table 2) with the exception of insulin treatment started after 13–24 months characterized by the highest seroreactivity; after splitting of patients according to LADA subtypes, LADA1 subjects showed Abs prevalence increasing with length of insulin-free periods from 14.28% to 25%, while opposite trends were observed for LADA2 decreasing from 18.75% to 10% of seropositive individuals. However, a few positive cases in each period do not permit to draw significant conclusions.

Although sex-related analysis did not reveal associations with high titers of Abs against the selected peptides, the number of males and females varied among LADA phenotypes based on patients’ age at onset. Men accounted for 60% of LADA1 subjects between 32 and 51 years old, whereas proportions in older groups were close to 1:1. In contrast, individuals assigned as LADA2 and aged 42–51 or older than 61 years at diagnosis were characterized by 1:2 male to female ratio.

## Discussion

We here demonstrated the increased prevalence of Abs targeting a portion belonging to a MAP-derived putative regulator for proline utilization (MAP2404c) and its human PI homolog with high sequence identity corresponding to contiguous fragments of B chain and C-peptide in Sardinian LADA patients. The study cohort was recruited from the local population known for an elevated incidence of autoimmune diseases [25] that has previously shown a significant reactivity against glucan branching protein of MAP (MAP1,4-αgbp) and the homologous peptide derived from B chain of human PI in subjects suffering from Hashimoto’s thyroiditis [26]. Likewise, responses to the same epitope pairs were even higher in Sardinian infants at risk for T1D [19] and new-onset children from mainland Italy [20]. In a similar fashion, increased seroreactivity to ZnT8 transmembrane regions and their homologous MAP peptides (MAP3865c) has been observed in either Sardinian or Italian cohorts at different disease stages [27, 28, 29, 30]. These results are in line with the ubiquitous presence of MAP in the environment and the frequency of exposure resulting in a straightforward transmission pathway to humans. Detection of Abs may indicate past contact with MAP following to consumption of contaminated food; most likely the intracellular form of MAP acquires enhanced virulence for humans after passage through bovine macrophages present in milk and cheese [31]. On the other hand, raised anti-MAP Abs titers have been found in individuals at continual interaction with infected animals such as veterinarians or livestock breeders; seropositivity among HCs and subjects at-risk for diabetes who do not develop clinical symptoms can be explained by exposure in early childhood to the extracellular phenotype of MAP that may confer natural immunity protective against mycobacterial infection [32].

Unlike T2D patients who do not display significant anti-MAP responses [21], LADA2 was characterized by similar Abs titers as LADA1 subtype. This outcome confirms the hypothesis involving MAP in the pathogenesis of autoimmune diabetes and sustains distinct mechanisms underlying the development of LADA2 with prevailing autoimmune component despite etiological factors shared with T2D. Among genetic risk factors, single nucleotide polymorphisms (SNPs) in *TCF7L2*, resulting in increased expression of the transcriptional factor, have been linked to T2D and LADA phenotype with low GADA titers [33, 34], whereas other authors have not reported any association [35, 36]. Interestingly, *TCF7L2* down-regulation has been observed in MAP-infected bovine monocyte-derived macrophages [37]. Data relative to *TCF7L2*-conferred risk were not available for our study cohort, however a future assessment based on the combination of these two factors in Sardinian population, considered its genetic homogeneity [38], may provide additional elements to complete our thesis.

Age-related analysis showed major prevalence of anti-MAP positivity for all the eight peptides in the youngest group (32–41 years) of LADA patients assessed either regardless or depending on GADA titers. When only the significant MAP/PI epitope pair was envisaged, this trend switched to the group over 61 years old for LADA2, while remained unvaried for LADA1 and for the general study population, even though the latter presented high Abs titers also in older groups. It is plausible that anti-mycobacterial Abs remain detectable at high levels even after 20 years following LADA diagnosis in a similar way to GADA titers [39], indicating a possible appearance of their peak values at a younger age. In addition, we have previously observed a comparable distribution of Abs targeting the homologous peptides in case of Hashimoto’s thyroiditis with distinct sex-related prevalence [26]. In the present study, responses to MAP antigens were not gender-associated, however Abs positivity in predisposed subjects, especially those assigned as LADA1, may suggest an increased risk for autoimmune thyroiditis. Comorbidity of both diseases has been already described and high GADA titers in males seem to be predisposing for thyroid autoimmunity [40, 41]. Moreover, males aged 32–51 years at disease onset accounted for 60% in LADA1 subgroup reflecting findings observed in case of T1D that confirm male excess in populations with high incidence such as Sardinia [42]. Male to female 1:2 ratio among LADA2 subjects was similar to distributions described for T2D.

We further assessed patients’ reactivity to the selected peptides in correlation with time interval from LADA diagnosis to insulin dependence. Abs prevalence relative to the significant MAP/PI epitope pair was distributed homogeneously among subjects who showed insulin-free period from 18 months to 4 years (within the range 17–18%); the percentage was slightly higher among patients requiring insulin treatment between 13–24 months (23.53%) and much lower in those without therapy after 4 years from LADA onset (14.28%). Surprisingly, after separated analysis of the two disease phenotypes, the decreasing anti-MAP Abs positivity trend was maintained for LADA2 subgroup and showed an inverted picture among LADA1 individuals. These results may suggest a possible association of MAP with a more severe disease course in LADA2 phenotype leading to a sooner need for insulin dosage, however a small number of patients assigned to each insulin-free period did not permit to obtain statistical significance. Moreover, no relation with GADA titers has been found in our previous studies; in contrast, MAP/PI and MAP/ZnT8 positivity has been correlated with respective classical T1D autoantibodies [19] not available for the present cohort. A deeper insight should be dedicated to this question given the increased anti-MAP responses detected among IA-2A-positive subjects.

Our work provides evidence of increased prevalence of antibodies directed against MAP epitope and its homolog derived from human proinsulin in LADA patients. Furthermore, it is in line with our previous findings highlighting elevated positivity to MAP in pediatric and adult populations with other autoimmune disorders such as T1D at different stages of β-cell immunity and Hashimoto’s thyroiditis. Further study is needed to address genetic predisposition in correlation with anti-MAP reactivity and the presence of non-islet organ-specific autoantibodies as a possible indication of comorbidities. Yet, information relative to risk factors in dietary habits may be useful in determining any correlation of LADA with sources of putative exposure to MAP.
